# Identification and characterization of differentially expressed miRNAs in subcutaneous adipose between Wagyu and Holstein cattle

**DOI:** 10.1038/srep44026

**Published:** 2017-03-08

**Authors:** Yuntao Guo, Xiuxiu Zhang, Wanlong Huang, Xiangyang Miao

**Affiliations:** 1 Institute of Animal Sciences, Chinese Academy of Agricultural Sciences, Beijing, 100193, China

## Abstract

MicroRNAs (miRNAs) are important post-transcriptional regulators involved in animal adipogenesis, however, their roles in bovine fat deposition remain poorly understood. In the present study, we conducted a comparative RNA sequencing to identify the key miRNAs involved in beef lipid accumulation by comparing the backfat small RNA samples between Wagyu (high intramuscular fat) and Holstein (moderate intramuscular fat) cattle. Fifteen miRNAs such as bta-miR-142-3p, bta-miR-379, bta-miR-196a, bta-miR-196b, bta-miR-30f and bta-miR-2887 were identified to have a higher expression level in Wagyu cattle compared with Holstein, whereas bta-miR-320a, bta-miR-874 and bta-miR-1247-3p had a lower expression level in Wagyu. Furthermore, a total of 1345 potential target genes of differentially expressed miRNAs were predicted using bioinformatics tools, in which PPARα and RXRα were known to play a critical role in adipocyte differentiation and lipid metabolism. In conclusion, the present study constructed a high-throughput RNA sequencing screen and successfully identified miRNAs such as bta-miR-874, bta-miR-320a and bta-miR-196b which may affect beef fat deposition. The present findings may provide a theoretical foundation for the utilization of beef cattle germplasm resources.

The subcutaneous and intramuscular fat deposits are important characteristic evaluations of cooked beef products. Marbling, identified as intramuscular fat content, contributes to meat tenderness, juiciness, and taste, which are all important for beef quality. Generally, subcutaneous tissue has priority for being filled out by adipocytes, followed by intramuscular areas, leading to less marbling deposition. It remains a big challenge to reduce subcutaneous fat and improve intramuscular lipid accumulation in modern beef production^1^. Adipogenesis, the process where pre-adipocytes differentiate into adipocytes, plays a crucial role in animal adipose accumulation^2^,^3^. A precise understanding of the molecular mechanisms of beef fat deposition is essential for the production of high quality cooked beef.

In recent years, there has been an increase in utilization of deep sequencing of the transcriptome for the identification of differentially expressed miRNAs as well as for the opportunity to discover novel transcripts, including new alternative isoforms and miRNAs^4^,^5^,^6^,^7^. With the rapid development of next generation sequencing (NGS), the study of miRNA becomes more attainable than before^8^,^9^,^10^,^11^. Fat deposition is a complex biological process where miRNAs may play a regulatory role. In adipose tissue, accumulating evidences clearly demonstrate that miRNAs play an important role in adipocyte differentiation and lipid metabolism^12^,^13^. It was found that many miRNAs such as let-7^14^, miR-143^15^,^16^,^17^, miR-17–5p^18^, miR-14^19^ and miR-33^20^,^21^,^22^ regulate the adipocyte differentiation and lipogenesis via various signaling pathways including Wnt, MAPK, cell cycle regulation and insulin pathway. Nuclear receptors like CCAAT/enhancer-binding proteins (C/EBPs), Peroxisome proliferator-activated receptor (PPARs) and Sterol-regulatory element binding proteins (SREBPs) are also important. These findings imply the miRNAs may take part in cattle lipid accumulation in adipose tissue and muscle.

It have been proved to be a feasible strategy to explore the mechanisms of adipogenesis and adipose accumulation by comparing the miRNA and/or mRNA expression patterns between different cattle breeds. Wang *et al*.^23^ compared the gene expression pattern of intramuscular fat between crossbreeds of Wagyu × Hereford and Piedmontese × Hereford, and a set of adipogenesis and lipogenesis related genes, such as Adiponectin (ADIPOQ), Stearoyl-CoA desaturase (SCD) and Thyroid hormone-inducible hepatic protein (THRSP), were up-regulated in the Wagyu × Hereford group^23^. Also another study compared the miRNA expression patterns of subcutaneous adipose tissue from several crossbred steers^24^. A comparison of purebred cattle with different fat deposition characteristics will provide some new information on the roles of different miRNAs.

Wagyu cattle are famous for their high marbling, i.e. a high level of intramuscular lipid accumulation, whereas another well-known beef cattle breed, Holstein cattle, has much less marbling compared to Wagyu^25^,^26^. The difference of fat deposition between Wagyu and Holstein cattle attracts lots of attentions for the comparative mechanism research in the field of beef adipogenesis and lipogenesis. Previous studies on the fat deposition difference between Wagyu and Holstein cattle are mainly focused on the differential expression of specific genes such as SCD^27^ and preadipocyte factor 1 (Pref-1)^25^. Until now, the difference of miRNA expression patterns between Wagyu and Holstein beef remains unknown. In the current study, a high-throughput sequencing screen was conducted to compare the miRNAs expression levels of subcutaneous adipose tissue between Wagyu and Holstein. We aim to identify possible miRNA regulators of adipose accumulation, providing new insights on the possible mechanisms of beef fat deposition.

## Results

### Construction of small RNA libraries

**Figure 1:**
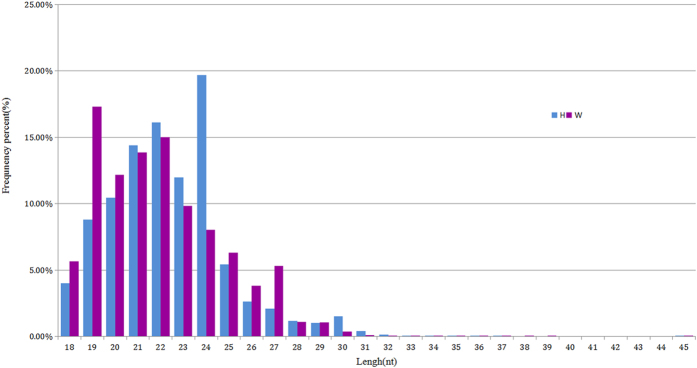
Frequency distribution of sequence lengths of the sequencing results in the W (red) and H (blue) libraries.

**Figure 2:**
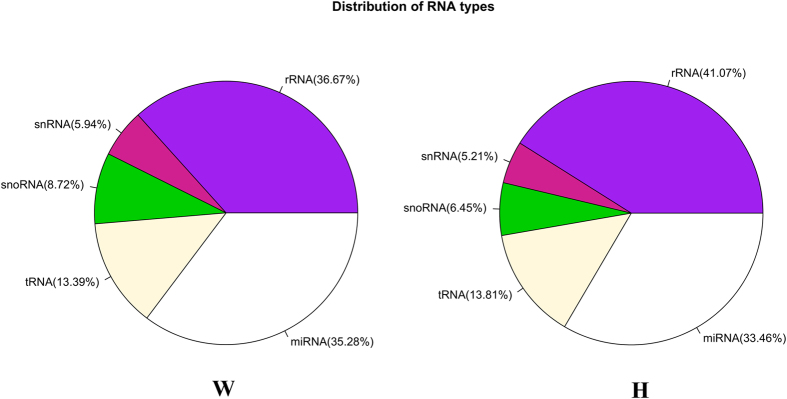
Percentage of various ncRNA reads in total distinct reads.

To identify the adipose differentially expressed miRNAs in backfat between Wagyu (W) and Holstein (H) cattle, we constructed W and H small RNA libraries. Using Illumina sequencing, a total of 12,435,335 and 10,962,269 raw reads were obtained from the W and H libraries respectively. After low-quality sequences, polyA, sequences shorter than 18 or longer than 45 nt, and repeated sequences were removed, 10,974,628 (88.32%) and 9,417,372 (85.96%) clean reads in the W and H libraries were finally obtained for further analysis, respectively (Table 1). The small RNA length distribution of the two libraries showed that the most abundant species were 21–22 nt in length, a typical size range for Dicer-derived products (Fig. 1). Then, these clean reads were aligned against Rfam database to filter the non-coding RNAs such as tRNAs, rRNAs, snoRNAs and snRNAs. A total of 35.28% and 33.46% distinct reads of the total small RNAs in W and H libraries were identified as conserved miRNAs, which indicates that the sequencing in the present study was successful (Fig. 2).

**Figure 3:**
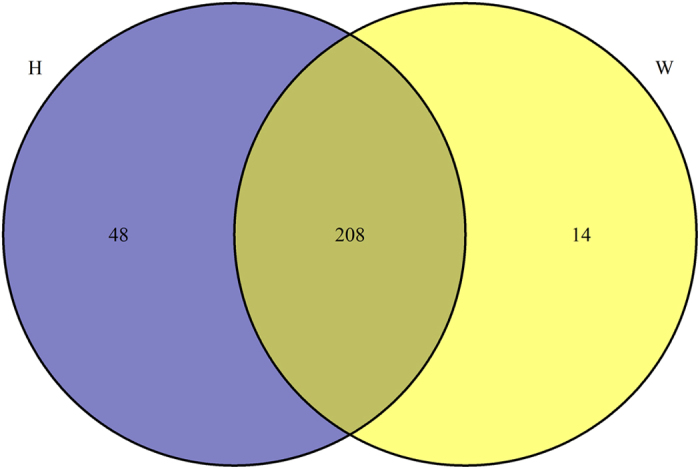
Venn diagram of conserved miRNAs in both W and H breeds.

**Figure 4:**
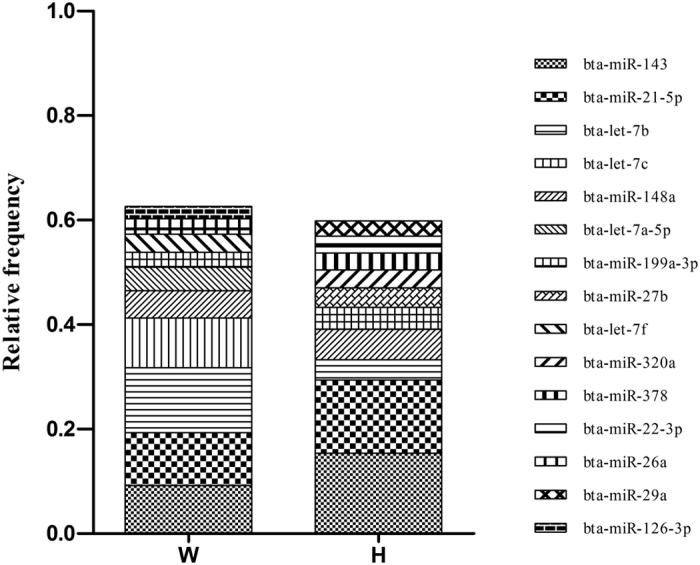
The top ten abundant miRNAs identified in the W and H libraries.

Further, the filtered reads were aligned against bovine genome, and the mapped miRNA sequences were selected to BLAST against miRBase to identify conserved miRNAs and calculate their expression levels. Totally 208 conserved miRNAs were successfully identified in both W and H libraries. Besides, 48 miRNAs were specifically expressed in backfat from Holstein cattle, and 14 miRNAs were only expressed in Wagyu cattle (Fig. 3). The sequences that did not match the conserved miRNAs were used to potentially predict new miRNAs. The transcripts per million (TPM) value analysis demonstrated that in the libraries W and H, the top ten high abundant miRNAs accounted for 60% of the total miRNAs, including let-7^14^, miR-143^15^,^16^,^17^, miR-21–5p^28^, miR-27b^29^,^30^,^31^ and miR-378^32^ which are previously reported to be involved in adipocyte differentiation (Fig. 4).

### Differentially expressed miRNA analysis between Wagyu and Holstein cattle

In the present sequencing study, the expression level of miRNAs which were expressed in the both libraries were quantified by TPM, if TPM with |log2FC| ≥ 1 and FDR adjusted p-value < 0.05, the miRNAs were considered as differentially expressed. In our study, 18 miRNAs were found with differential expression levels between W and H libraries, 15 had a higher expression level in W, whereas 3 in H (Table 2). Among them, the more expressed let-7, miR-142, miR-196b and lower expressed miR-320a have been reported to regulate lipid metabolism. Worthy of mention, miR-320a was in the top ten within the high abundant miRNAs, which implies that it may play an important role in beef adipogenesis.

### Novel miRNAs prediction

One important characteristic of miRNA is the hairpin structure, which can be used for new miRNA prediction. In the present study, the small RNA reads with no known pre-miRNA homologs in miRBase alignment were subjected to new miRNA prediction analysis of the secondary structure, the Dicer cleavage site and the minimum free energy using miRDeep2. In library W, 11 pre-miRNAs with stable hairpin structure were predicted, and 7 were predicted in library H. Among them, only one miRNA was expressed in both libraries.

### Target prediction of the differentially expressed miRNAs

**Figure 5:**
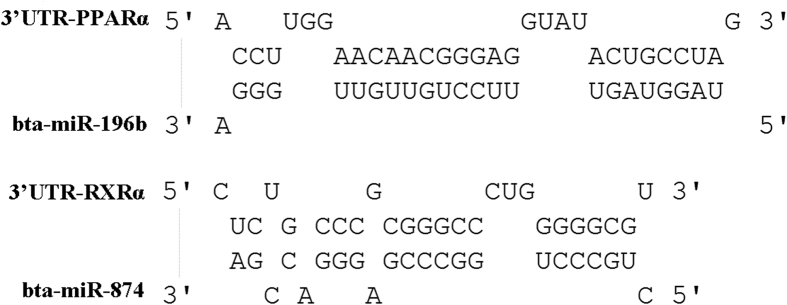
Bta-miR-196b, bta-miR-874 and their predicted target genes PPARα, RXRα.

Further identification of miRNA targets will help illustrate their functions of the differentially expressed miRNAs identified in the present study. The aligned genome sequences of miRNAs were then used for the prediction of target genes, and a total of 1345 genes were identified in current study as potential targets of the 18 differentially expressed miRNAs between library W and H. Among them, Apolipoprotein A-1 (APOA1), Apolipoprotein A-5 (APOA5), Angiopoietin-related protein 4 (ANGPTL4), Peroxisome proliferator-activated receptor alpha (PPARα), Retinoic acid receptor RXR-alpha (RXRα) and Cyclin-dependent kinase 11B (CDK11B) were thought to be involved in adipocyte differentiation and lipid metabolism. Particularly, two important transcription factors in PPAR signaling pathway, PPARα and RXRα, were also predicted to be the targets of bta-miR-196b and bta-miR-874, respectively (Fig. 5).

### GO and KEGG pathway annotation of miRNA target genes

Moreover, the 1345 predicted target genes were classified according to GO annotations using DAVID^33^. In the biological process category, a total of 75 GO terms were significantly enriched. Among them, the major enriched GO terms of target genes included fatty acid metabolic process, regulation of multicellular organism growth, glycerolipid metabolic process, triglyceride metabolic process, and regulation of cell cycle. Many of these processes were associated with lipid metabolism and adipocyte differentiation. In the molecular function category, the predicted target genes were classified in 16 GO terms with significance, including protein serine/threonine kinase activity and lipid binding within the most importants. The cellular component category showed that the target genes were enriched in 18 significant GO terms, most of them were related to mitochondria, suggesting that the target genes may be involved in the regulation of energy metabolism. Furthermore, the predicted target genes were annotated in KEGG pathways to identify potential pathways that may be regulated by the differentially expressed miRNAs. The results indicated that target genes were enriched in significant pathways like glycerophospholipid metabolism, Notch signaling pathway and PPAR signaling pathway which have been reported to be closely related with adipogenesis^34^.

### miRNA-protein interaction analysis

**Figure 6:**
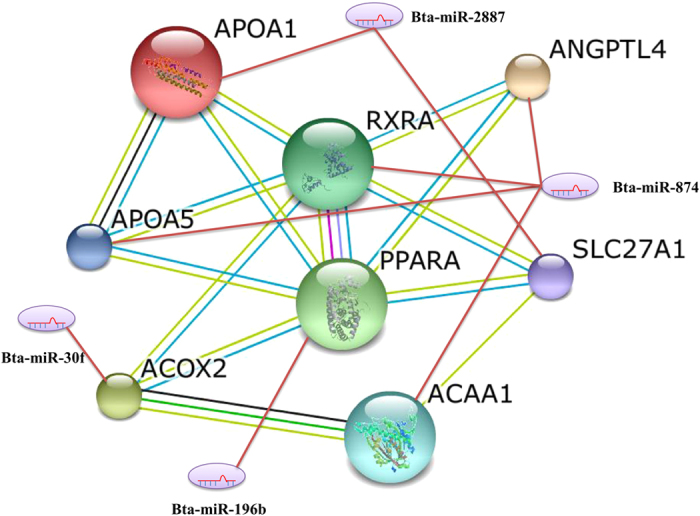
Interactive relationship between differentially expressed miRNAs and their target genes in PPAR signaling pathway.

A miRNA-protein interaction network was constructed to reveal the relationship of predicted target genes in PPAR signaling pathway, and miRNAs were led in to identify the potential regulators (Fig. 6). Consistent with the data presented in Fig. 5, the results illustrated that PPARα and RXRα played a central role in PPAR signaling pathway, and they might be targets regulated by bta-miR-196b and bta-miR-874, respectively.

### Validation of miRNA sequencing by qPCR

**Figure 7:**
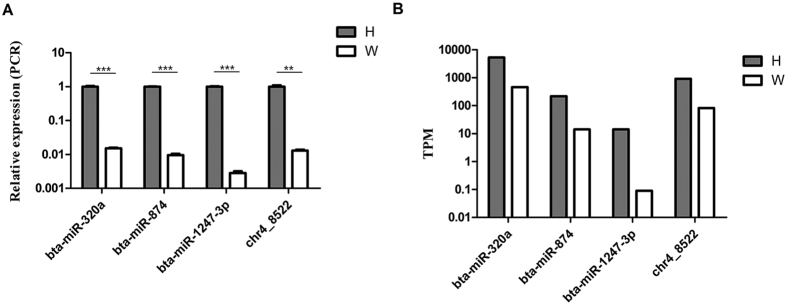
Comparison of relative expression (**A**) and TPM (**B**) values of four selected differentially expressed miRNAs. (**A**) The relative expression level of selected miRNAs were measured by quantitative real-time PCR, data were expressed as the means ± SD. **, ***p < 0.01, or 0.001, respectively. (**B**) The TPM of selected miRNAs analyzed in RNA-seq.

The expression level of 4 differentially expressed miRNAs were selected and verified by qRT-PCR (Fig. 7). Consistent with the miRNA sequencing data, the qRT-PCR results of the 4 randomly selected miRNAs showed a similar pattern of expression in the two libraries, which further confirmed that our sequencing data were reliable.

## Discussion

The intramuscular fat contributes to beef quality, and it is a big challenge to reduce subcutaneous fat and increase intramuscular fat. More complete understanding of fat deposition mechanism is vital for beef cattle breeding and beef production. MicroRNAs are important post-transcriptional molecules regulating adipogenesis and lipid accumulation as previously discovered^35^. However, their precise roles of miRNAs in bovine fat deposition are far from clear. In the present study, we conducted a high-throughput RNA sequencing to identify miRNAs associated with the different fat deposition characteristics between Wagyu and Holstein cattle. In the present study, we found that the expression levels of miR-142-3p, miR-379 and miR-196a were higher in the adipose tissue of Wagyu cattle compared with Holstein (Table 2). Consistently, the studies from Chartoumpekis *et al*. (2012) and Meale *et al*. (2014) have also reported that these miRNAs regulate lipogenesis and fat deposition in other animals^36^,^37^. Taken together, the present study indicates that the higher intramuscular fat level in Wagyu cattle may be partially attributed to these 3 highly expressed miRNAs.

Interestingly, bta-miR-320a was also presented in the top ten high abundant miRNAs, and it exhibited a significantly differential expression pattern between Wagyu and Holstein cattle (P < 0.05). MiR-320 family have been found to promote adipocyte differentiation via inhibiting Runt-related transcription factor 2 (RUNX2) which is a key osteoblast-specific transcription factor that induce mesenchymal stem cells (MSCs) to differentiate into osteoblasts and suppress adipocytic differentiation^38^. MiR-320 family promote adipogenesis via blocking other MSCs differentiation pathways (i.e., osteoblast). Another study also revealed that miR-320 regulates insulin resistance via insulin-PI3K signaling pathway^39^. Taken together with our present result, it may be concluded that miR-320 plays an important role in adipogenesis and subsequent difference in fat deposition between Wagyu and Holstein cattle.

Moreover, we found that the expression levels of miR-136, miR-190a, miR-411a, miR-708, miR-1940 and miR-2887 were significantly higher in Wagyu compared with Holstein cattle (P < 0.05), while the expression levels of miR-874 and miR-1247–3p were lower (P < 0.05). To the best of our knowledge, it is the first time these miRNAs were reported involved in regulation of adipose tissue and their roles in fat deposition are not clear, which deserve to be further investigated in the coming future.

The GO annotation illustrated that the predicted target genes of differentially expressed miRNAs were mainly classified in biological processes related to fatty acid metabolic process, regulation of multicellular organism growth, glycerolipid metabolic process, triglyceride metabolic process, and regulation of cell cycle. Interestingly, KEGG pathway annotation of target genes identified three adipocyte differentiation and lipid metabolism related pathways, including glycerophospholipid metabolism, Notch signaling pathway and PPAR signaling pathway. Glycerophospholipid metabolism is an important part of organism phospholipid metabolism. bta-let-7c, bta-let-7f, bta-let-7a-5p and bta-miR-196b might regulate glycerophospholipid metabolism by inhibiting the expression of enzymes such as Acyl-protein thioesterase 2 (LYPLA2), Phospholipase A2 (PLA2G4B) and Diacylglycerol kinase (DGKH), which have been found to take part in the synthesis and degradation of triglycerides^40^. Notch signaling pathway is a crucial pathway in ontogenesis and cell differentiation^41^. Several previous studies have indicated the role of Notch pathway in pre-adipocyte differentiation, and pointed out that the activation of Notch signaling pathway may inhibit the differentiation of adipocytes^42^,^43^,^44^,^45^. In the current study, 8 miRNA target genes including Delta-like protein 1 (DLL1), Segment polarity protein dishevelled homolog DVL-3 (DVL3), Histone acetyltransferase KAT2A (KAT2A), Gamma-secretase subunit PEN-2 (PSENEN), Transcription factor HES-5 (HES5), Histone deacetylase 1 (HDAC1), E3 ubiquitin-protein ligase DTX4 (DTX4) and E3 ubiquitin-protein ligase DTX2 (DTX2) were involved in Notch signaling pathway, suggesting that some miRNAs regulate adipocyte differentiation via Notch pathway.

Interestingly, the PPAR signaling pathway was highly enriched in the present study (Fig. 6). Recent studies have demonstrated the critical roles of PPARs in lipid and carbohydrate metabolism, cell differentiation, growth, apoptosis and inflammation^46^,^47^. It was illustrated that bta-miR-30f, bta-miR-196b, bta-miR-874 and bta-miR-2887 could regulate the lipid metabolism, glycerophospholipid metabolism, adipocyte differentiation and glucose metabolism via inhibiting the expression of PPAR pathway related genes such as PPARα, RXRα, 3-ketoacyl-CoA thiolase (ACAA1), Apolipoprotein A-5 (APOA5), Peroxisomal acyl-coenzyme A oxidase 2 (ACOX2), Angiopoietin-related protein 4 (ANGPTL4), Apolipoprotein A-1 (APOA1), Integrin-linked protein kinase (ILK), Ubiquitin-60S ribosomal protein L40 (UBA52) and Long-chain fatty acid transport protein 1 (SLC27A1) (Fig. 6). Particularly, bta-miR-196b and bta-miR-874 directly regulate the expression of PPARα and RXRα respectively (Fig. 5). Both PPARα and RXRα play a central role in PPAR pathway. As is proved, PPARγ and C/EBPs are key transcription factors in regulating adipogenesis, but in our study, PPARγ wasn’t identified as any targets of differentially expressed miRNAs. Furthermore, PPARα, which is also a core element in PPAR pathway, is signally differentially expressed. In the study of obesity, the up-regulated miR-519d could promote the lipid accumulation in preadipocyte through PPARα^48^. In the current study, the expression of bta-miR-196b was found to be higher in Wagyu cattle than in Holstein, implying that it may regulate fat deposition in beef via stimulating adipocyte differentiation through the PPAR pathway like miR-519d. Another critical functional element in the PPAR pathway, RXRα, regulates the transcription by binding the promoter RARE region of target genes, regulating cell differentiation^49^. In Holstein cattle fed long-chain fatty acids, PPARγ and RXRα form a heterodimer to modify the gene expression in adipose tissue^50^. Taken together, the present study clearly demonstrated the differentially expressed miRNA patterns in subcutaneous adipose tissue between Wagyu and Holstein cattle. The present findings may provide a theoretical foundation for the utilization of beef cattle germplasm resources.

## Conclusion

In the present study, we used a high-throughput small RNA sequencing to compare the miRNAs level of backfat between Wagyu and Holstein cattle. Based on our knowledge, this is the first study about miRNA profiles of adipose tissues between Wagyu and Holstein cattle to explore the possible mechanisms of fat deposition. In our study, several miRNAs are identified which may regulate adipogenesis through their targets and related pathways. Among them, up-regulated bta-miR-196b and down-regulated bta-miR-874 may influence the signal translation of PPAR pathway by their targets involved in the pathway and, consequently, regulate fat deposition. Our results provide information about microRNAs that were differentially expressed in subcutaneous fat of two different beef breeds with different levels of marbling deposition, providing a better understanding of the molecular mechanisms of beef fat deposition.

## Materials and Methods

### Experimental animals and sample preparation

All experiments were performed in accordance with relevant guidelines and regulations is sued by the Ministry of Agriculture of the People’s Republic of China. All experimental protocols were approved by Institute of Animal Sciences, Chinese Academy of Agricultural Sciences where the experiment was conducted.

In the present study, the Wagyu and Holstein cattle were provided by Beijing Huairou Wagyu Technology CO. (Beijing, China) and Langfang Xingcheng meat production company (Hebei, China), respectively. Five male Wagyu and five male Holstein cattle around 30-months old were selected to collect the dorsal subcutaneous adipose tissue between 12th and 13th rib. The adipose tissue were collected right after animals were sacrificed, and immediately stocked in liquid nitrogen for further RNA extraction.

### Small RNA library construction and sequencing

Total RNAs were extracted from the adipose tissue using TRIzol (Invitrogen, USA) according the manufacturer’s instructions. Aliquots of the total RNA (10 μg) from five Wagyu or Holstein cattle were mixed in equal amounts to generate a pooled sample as the library W (for Wagyu cattle) and H (for Holstein cattle) respectively. The total RNA concentration was measured by NanoDrop 2000 (GE system, USA) spectrophotometer and Agilent 2100 bioanalyzer (GE system, USA). Small RNAs were isolated using PEG-8000 (Sigma, USA), RNAs were ligated with 3′ adapter, and then size fractionated by 15% denaturing polyacrylamide gel electrophoresis. Fractions of 36–44 nt were recovered and ligated to 5′-adapter. The small RNA fractions were reverse transcribed and then amplified by PCR, the amplicons were loaded into 3.5% agarose gel, fractions of 140–160 bp were recovered as library. The small RNA libraries were checked by qPCR and the concentration were over 2 nM, indicating that the libraries were reliable. Then the libraries W and H were sequenced using Hiseq-2000.

### Bioinformatics analysis of small RNA sequences

Raw reads obtained from the libraries were first treated by Cutadapt and FASTX-toolkit softwares to get clean reads in FASTQ files. The quality assessment of sequencing data, including base distribution, GC ratio, PCR duplication and frequency of kmer, were valued by FastQC software. The FASTQ Information function in FASTX-toolkit was used for RNA sequence length distribution analysis. Clean small RNA sequences ranging between 18 and 45 nt in length obtained from the libraries were aligned against Rfam database (Rfam 10.0) using Rfamscan and Blastn to remove rRNAs, scRNAs, snRNAs, snoRNAs and tRNAs^51^. The rest of distinct sequences were used to search in miRBase (miRBase19.0) using miRDeep2 to identify the conserved miRNAs^52^,^53^. To predict new miRNA candidates, the sequences were mapped in the bovine genome from ftp://ftp.ensembl.org/pub/release-76/fasta/bos_taurus/dna/ using bowtie2 and calculated by miRDeep2.

### Differentially expressed miRNA identification and target prediction

The counts of conserved miRNAs identified from miRDeep2 between W and H libraries were used for expression analysis^54^. In order to assess the significance of the miRNA expression difference, the R package “edgeR”^55^ program was used to compute the counts data set. Worthy of mention, to avoid false positive results, only TPM with |log2FC| ≥ 1 and FDR adjusted p-value < 0.05 were identified as differentially expressed miRNAs.

To predict the target genes of differentially expressed miRNAs, software RNAhybrid was used, with the parameters e ≤ −20 and P < 0.01^56^.

### Gene Ontology (GO) and Kyoto Encyclopedia of Genes and Genomes (KEGG) pathway annotation of miRNA target genes, and miRNA-protein interaction analysis

The GO analysis of screened miRNA target genes was performed to predict the potential biological processes and functions that were most likely to be affected by miRNAs using Database for Annotation, Visualization and Integration Discovery (DAVID)^57^. Top significant GO categories, biological functions and different canonical pathways were analyzed for miRNA specific targets as well as for all screened targets based on significant over-representation of genes using Fisher test. Then the miRNA target genes were annotated in KEGG pathway using DAVID^58^. Additionally, we applied String database (http://string-db.org/) to predict the miRNA-mRNA target gene interaction network.

### qPCR validation of identified miRNAs

The expression level of 3 differentially expressed miRNAs, bta-miR-320a, bta-miR-874 and bta-miR-1247-3p, and a predicted novel miRNA chr4_8522, were randomly selected and verified by qRT-PCR. Briefly, total RNAs extracted from cattle backfat were reversely transcribed to get cDNA. Then real-time PCR reaction was performed to measure the miRNA expression levels. All reactions were performed in triplicates with reaction volume of 10 μl (5 μl of 2 × LightCycler 480 SYBR Green I Master Mix, 0.2 μl of PCR forward primer, 0.2 μl of PCR reverse primer, 1 μl of cDNA and 3.6 μl of nuclease-free H_2_O) and incubated (95 °C for 10 min, then 40 cycles at 95 °C for 10 sec, 60 °C for 30 sec). The comparative Ct method and internal control miRNA gene U6 were used for the calculations of the relative expression levels of the genes. All of the qPCR reactions yielded a single peak on the dissociation curve, indicating specific amplifications.

### Statistical analysis

TPM was used to evaluate the miRNA expression levels and it was calculated according to the formula. In the formula, *librarysize* represents total counts of all miRNAs identified and *miR_readscounts* stands for reads number of the certain miRNA.


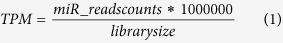


When processing target gene enrichment analysis, the hypergeometric distribution and fisher exact test was used to calculate p-values using the formula below. In the formula, *a* represents the number of target genes in the GO or pathway term to be detected, *b* stands for the non-target genes the number of non-target genes replaces the non-target genes number of the same term, *c* means the number of target genes in the other terms, while *d* means the number of non-target genes in the other terms. As it’s a multiple test, the p-values are adjusted by the FDR (false discovery rate) method.


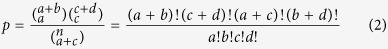


All data of qPCR are presented as the means ± SD. When comparisons were made, Student’s t-test was performed using SPSS 17.0 (IBM, USA) and p < 0.05 was considered statistically significant.

## Additional Information

**How to cite this article**: Guo, Y. *et al*. Identification and characterization of differentially expressed miRNAs in subcutaneous adipose between Wagyu and Holstein cattle. *Sci. Rep.* **7**, 44026; doi: 10.1038/srep44026 (2017).

**Publisher's note:** Springer Nature remains neutral with regard to jurisdictional claims in published maps and institutional affiliations.

## Figures and Tables

**Table 1:** Results of raw reads before and after quality control of the Wagyu (W) and Holstein (H) libraries.

Reads Type	W		H	
	Reads Number	Ratio	Reads Number	Ratio
Total reads number	12435335	100%	10962269	100%
Low quality	9594	0.08%	6166	0.06%
Adaptor 3 null	130848	1.05%	118868	1.08%
Insert null	44218	0.36%	149945	1.37%
5′ adaptor contaminants	16573	0.13%	66645	0.61%
Size < 18 nt	1258706	10.13%	1200889	10.96%
PolyA	768	0.01%	2384	0.02%
High quality (size ≥ 18 nt)	10974628	88.32%	9417372	85.96%

*The first column means the reads types raw reads are processed to obtain clean reads, Reads Number means the reads (tags) number of each type in W and H libraries, Ratio means the fractional reads (tags) number of each type in total reads.

**Table 2:** Differentially expressed known miRNAs identified in the W and H libraries.

miRNA name	log2FC	FDR	H_TPM	W_TPM
bta-miR-196b	7.2253	0	2.6547	227.4337
bta-miR-1940	10.2224	0	0	10.8432
bta-miR-196a	4.9385	0.0001	12.8486	224.5179
bta-miR-2487	5.0199	0.0001	2.0175	37.5411
bta-miR-1247-3p	−6.3596	0.0003	14.4414	0.0911
bta-miR-142-3p	3.7759	0.0041	8.3888	65.5147
bta-miR-136	3.6343	0.0070	4.8846	34.6253
bta-miR-2887	3.3692	0.0134	10.0877	59.4098
bta-miR-411a	3.4559	0.0146	1.9114	12.0277
bta-miR-379	3.2691	0.0146	8.3888	46.1063
bta-miR-708	3.2481	0.0146	79.5339	430.1740
bta-miR-874	−3.1297	0.0177	220.1251	14.3057
bta-let-7c	3.1175	0.0177	1676.2638	8279.8251
bta-let-7f	2.9105	0.0326	701.1510	3000.5573
bta-miR-30f	3.0376	0.0345	3.3980	15.9459
bta-let-7a-5p	2.8427	0.0360	951.2208	3883.8674
bta-miR-190a	2.9183	0.0463	2.6547	11.4810
bta-miR-320a	−2.7345	0.0463	5366.7839	458.9677

*log2FC means log_2_(W_TPM/H_TPM), FDR is equal to false discovery rate, W_TPM stands for transcripts per million for Wagyu, H_TPM represents transcripts per million for Holstein.
